# Simplicity and predictability: a phenomenological study of psychological flow in transactional workers

**DOI:** 10.3389/fpsyg.2023.1137930

**Published:** 2023-06-02

**Authors:** Steven R. Clapp, Waldemar Karwowski, P. A. Hancock

**Affiliations:** ^1^Department of Industrial Engineering and Management Systems, University of Central Florida, Orlando, FL, United States; ^2^Department of Psychology, University of Central Florida, Orlando, FL, United States

**Keywords:** psychological flow, motivation, work system factors, work system design, qualitative methods

## Abstract

Psychological flow is a positive experience achieved through a near-balance of task challenge and skill capability, creating a merging of awareness and action and leading to an intrinsically rewarding feeling. Flow has typically been documented in persons who participate in work and leisure activities where they can exercise a large degree of creativity and agency over their actions in pursuit of their goals. The objective of the present study is to explore the lived experiences of flow in workers in roles where creativity and agency are typically not expected. An interpretative phenomenological analysis approach was employed to attain this objective. Semi-structured interviews were conducted with 17 adults whose role is to perform transactional work, which by its nature affords less opportunity for creative execution. Common themes about participants’ flow experiences are documented. Two broad types of flow are described and a connection is made that the present study’s participants achieve one of those flow types while working. Participants’ feelings, preferences, and actions are mapped to the nine conventional dimensions of flow. Specific non-task work system factors are discussed relative to their influence on participants’ attainment of flow. Limitations of the present study and recommended future research are discussed.

## 1. Introduction

### 1.1. The flow experience

Psychological flow is the state where one is so immersed in an activity that all other sensations seem to fall away; the passage of time feels altered and even the sense of self is subordinated to the completion of the task. Csikszentmihalyi (1990) describes this state as an “optimal experience.” The feeling of being in flow at work has been described as being completely immersed in a rich and demanding task; of being in harmony with the work and the people working with you; of not wanting to stop what you are doing, even when tired; and of enjoyment and fun (Csikszentmihalyi, 2003), pp. 39–41. Greguras et al. (2014); p. 153 equate flow at work to what happens when one expends just the right amount of effort and engagement on a task of sufficient challenge; when the skills of the person fit the challenge of the job, employees are absorbed in their work and autonomous motivation occurs.

According to Csikszentmihalyi (1990) and adopted by others (for example, Jackson and Marsh, 1996), flow consists of nine dimensions. These dimensions are time-sequenced (Csikszentmihalyi, 1988; Chen et al., 1999; Fullagar et al., 2017), such that antecedent dimensions lead to characteristic dimensions, which in turn lead to consequential dimensions. Antecedents form the foundation of the flow experience and must be initially present for flow to occur. Challenge/skill balance is considered the most critical antecedent (LeFevre, 1988; Csikszentmihalyi, 1990; Bakker, 2008; Keller and Landhäußer, 2012). If the challenge of the work, hobby, or chore task just exceeds the skill level of the one performing the task, flow is more likely to occur. However, if the challenge far exceeds skill level, worry and anxiety are more likely to be the actor’s mental state. And if the challenge is far below the individual’s skill level, apathy and boredom are apt to occur.

The second antecedent is having a clear goal to accomplish. This flow dimension answers the questions, “Do I know why am I performing this task?,” “Can I expect to execute this task well, in terms of the goal to be achieved?,” and “Does this goal align with my own values?” These questions relate to understandability, achievability, and relatability, respectively. Positive-oriented answers are more likely to lead to high task engagement (Csikszentmihalyi, 2003; Clapp et al., 2018).

The third antecedent dimension is immediate feedback. Knowing if current performance is leading the actor toward or away from a goal will help the individual stay on track or course correct, respectively. The narrower the time gap between task execution and feedback, the faster the performer can know to maintain his/her level of performance or if some adjustment is needed and the more relevance that can be attached to it. Such performance information can be generated internally (such as comparing one’s own results to a standard or to previous results), from other individuals (for example, post-sale customer feedback surveys and daily Agile scrum team meetings), and by the organization (for example, employee performance monitoring systems; Hackman et al., 1975; Csikszentmihalyi, 2003).

Characteristic dimensions are those aspects that occur while one is experiencing flow. One of these dimensions is the merging of action and awareness. That is, an individual in flow seems to know without consciously thinking the correct steps to perform and in which order; action is described as taking place instinctively (Csikszentmihalyi, 1990, 2003).

Being completely immersed in deep concentration is a second feature of flow. All external stimuli fade away and even internal, physiological needs such as hunger and fatigue may not be recognized while in the state of flow (Csikszentmihalyi, 1990). Of course, the long-term effects of ignoring physiological signals can be deleterious to one’s health and may be a sign of behavioral addiction; for a deeper discussion of the dark side of flow (see Schüler, 2012).

A third characteristic of flow is the sense of control (or agentic power) experienced by the individual over how the task at hand is to be accomplished. Self-directed decisions such as the order of items to process, the speed at which to execute task steps, and when to take work breaks (Csikszentmihalyi, 2003). The authors of the present study will show later in this paper that even the arrangement of the work environment contributes to the feeling of control over how the work is performed.

As a consequence of these previous six dimensions, persons who have experienced flow comment that they experienced a loss of ego, of self-consciousness. Thoughts of how others might perceive their task execution fall away. Maybe that is why we see some musicians making odd facial expressions while they are playing a passage, seemingly oblivious to the audience’s perception of said contortions. Or why we may hear our workmates talking to themselves while working on a challenging task. The person disappears; only the activity and the performance remain (Csikszentmihalyi, 1990).

Another consequence of flow is a perceived alteration of the passage of time experienced by some. For example, individuals who have experienced flow have commented that they thought they had been immersed in a task for a short period of time when in fact a much longer period of time had elapsed. For others, the opposite is true (Csikszentmihalyi, 1990; see also Hancock et al., 2019, for a discussion on the relationship between flow components and the experience of the passage of time).

The third consequence of flow is the feeling of enjoyment associated with accomplishing or even making progress toward completing or mastering a task. Csikszentmihalyi (1990) describes this enjoyment stemming from an intrinsic reward mechanism (as opposed to receiving compensation, recognition, or some other external reward) as an autotelic experience. This internal reward system is what encourages individuals to return to the activity again and again. For a sample of activities provide individuals intrinsically rewarding experiences, see Chen and Chen, 2011; motorcycle riding at high speeds and Seger and Potts, 2012; video game playing to satisfy the need to learn.

Experiencing flow in the workplace has been linked to greater well-being, positive affect, and greater commitment to the organization (Bryce and Haworth, 2002; Eisenberger et al., 2005; Demerouti, 2006). Research has also correlated flow to increased worker productivity (Martin, 2005; Demerouti, 2006 for workers with high levels of conscientiousness).

### 1.2. Understanding flow from a holistic work system perspective within transactional work

Flow has been long studied in the context of work, hobbies, sports, and other domains where paths to attaining desired outcomes can take many forms. For example, university classroom teaching of a subject can be executed by instruction with a textbook, with instructor-developed slide presentations, with guided experimentation, with free-form exploration, or with any combination thereof. The instructor is able and may be expected to, within prescribed institutional guidelines, create the curriculum of his/her choosing based on his/her level of experience and expertise. In other words, the instructor has much agency over how the course outcomes are to be met (Csikszentmihalyi and LeFevre, 1989).

But what about those who must perform rote, repetitive work in an office setting (some of which are colloquially referred to as “cubicle farms”) with little opportunity for creative control over the execution of tasks or over outcomes? Think data entry clerks, accounts payable staff, and schedulers. These workers are in transactional roles (Workforce Management., 2007; Hunt, 2008; Power, 2012) and comprise over 18.5 million members of the U.S. workforce (Bureau of Labor Statistics., 2020). We label these individuals as “transactional workers” who perform “transactional work.” Can this large workforce also experience flow and enjoy its concomitant benefits?

We chose to perform a phenomenological study since our objective was to “understand [the] phenomenon [of flow] from the point of view of the lived experience in order to be able to discover the meaning of it” (Englander, 2012). Specifically, we chose to employ an interpretive phenomenology analysis to be able to map participants’ descriptions of their flow experiences to well-known dimensions of flow as described in the introduction section of this present paper (Smith and Osborn, 2004; Finlay, 2009). Our study took the form of semi-directed one-on-one interviews of 17 transactional office workers to document their lived experience of flow at work. These persons conduct their daily activities largely seated at a desk and perform their tasks via a computer and software. Most of their tasks are performed with no or limited interaction with internal or external customers. We attempted to understand what flow feels like to these individuals what triggers their immersion into flow, and what causes the feeling of flow to end. Our interpretive framework is the extent to which certain non-task work system factors influence their attainment of flow. Non-task work system factors and an illuminating example are documented below.

The work system theory of flow (Clapp et al., 2018) proposes that both the task and non-task variables must combine to create a flow-supportive environment. In the present study, non-task work factors identified for further study to support this theory include work-supportive technology, background noise presence, clarity of task communications, and physical environment. These variables were chosen for study as they are typically found in office environments.

Technology was defined for the current study’s participants as the total set of tools needed to complete or aid in completing their work tasks. Such tools could be electronic in nature, such as computer networks and photo-copiers, and non-electric, such as written work instructions and writing instruments.

Transactional work environments are also referred to as back offices and shared service centers. They are typically characterized by collocating workers in functional groups, the goal of which is to achieve work efficiencies and reduce expenses (for examples, see Howcroft and Richardson, 2012; Proite, 2020). Such locations can be populated by anywhere from one or two employees (as in the university department settings of some of the present study’s subjects) to hundreds sharing an open floor separated by low walls (colloquially known as a “cubicle farm,” as in the financial services firm of others in the present study). Although there can be many individuals sitting in proximity to each other, each is executing his or her own tasks alone, with typically little interaction with others.

A constant babble of background noise can typically be found in these environments. Talking, rustling paper, and playing recorded music are examples of such background noise. The authors wanted to explore the influence of the presence of co-workers and the background noise they generate on participants’ ability to achieve flow at work.

The present study defined task communication as the level of detail contained in instructions (1) given by a supervisor to the subject, regarding what tasks to perform, the priority of those tasks against each other, and/or how to perform them; or (2) found in training that taught and standard operating procedures that documented the step-by-step actions needed to complete the tasks. The authors wanted to gain insight into whether the quality of such communication affected the participants’ ability to achieve flow.

In addition to typically sitting in proximity to other workers with this factor’s attendant effects from noise, offices also include physical environment variables such as ambient temperature ranges, various lighting levels, noise from a number of non-human sources, and seating and work desk influences like height, depth, and comfort. This list is not comprehensive but serves to illustrate the variety of potential influences on the achievement of flow the present study’s authors wished to explore.

As a test of the work system theory of flow, a proof-of-concept empirical study was conducted to determine if and to what extent seat comfort was an influence on the attainment of flow. The study found that seat comfort is in fact a main effect predictor of flow (Clapp et al., 2021). In this experiment, an ergonomically adjustable chair was quantitatively found to contribute to a higher achievement of a flow state in participants than an armless, backless bench did, for participants performing a computerized set of tasks in an office setting.

## 2. Materials and methods

### 2.1. Participants

Purposive sampling was employed to select candidates for the study. Seventeen transactional workers were recruited through personal communications by one of the authors (SRC) of this present study and through social media. Screening criteria included confirmation in writing by the participants prior to their appointed interview time of (1) working in an office-type setting, performing repetitive, well-defined, and routine tasks (transactional work as defined by Workforce Management. (2007), Hunt (2008), and Power (2012); (2) working at least 20 h per week in the said role; (3) attaining at least 18 years of age at the time of the study; and (4) experiencing the following feeling while at work [adapted from Csikszentmihalyi, 1990 and Bakker, 2008]:

The task is so demanding and rich in its complexity and pull. I get so immersed in what I’m doing, in the actions that are involved, that I lose consciousness of my own body and melt into the activity. My daily work challenges me, but I feel I have the skills to excel at those tasks. I know exactly what actions I must do to complete my work. I clearly know if I am achieving success in my work. I am so immersed in my work that I am not concerned about how others perceive me. I don’t notice how much time passes during my work. I feel a sense of enjoyment from a job well done and want to experience that feeling again.

None of the candidates approached refused to participate or were disqualified because of the screening criteria. Of the 17 participants, 13 were female. The average age was 41.4 years (SD = 18.1 years); median age was 47 years, and interquartile range was 27 years. The average time in role was 7.5 years (SD = 8.7 years); median time in role was 4 years, and interquartile range was 6 years. Occupations included office/administrative assistant, office manager, bookkeeper, accounts receivable clerk, lab analyst, database entry clerk, quality assurance associate, production support specialist, and space utilization coordinator.

This research complied with the American Psychological Association Code of Ethics. The institutional review board of the University of Central Florida approved this research. Written informed consent was provided to each person who expressed interest in participating in the research. Each participant was compensated US $20 upon completion of his/her interview, in line with local average hourly wages for roles represented by the participants. We do not believe this amount of remuneration was of a size that would have biased the participants’ input. Duration of the interview sessions were approximately 45 min. No personally identifiable information was collected during the interview sessions to preserve subjects’ anonymity.

This being a qualitative study, no statistically valid sample size calculations were appropriate. Sampling was purposive and followed the guidance of Smith and Osborn (2004): the sample size was determined on an ongoing basis throughout the interview process, finally reached when the authors felt the topics had been saturated with respondents’ feedback and insights, and duplication of responses began occurring with some frequency.

### 2.2. Setting

The study was performed in person for roughly one-third of the participants and via video conferencing for the remainder. Solely the participant and the interviewer were present for each interview session. Interviews were conducted with fifteen of the individuals located in their offices; two were held away from the participants’ work locations due to the lateness of the day (one via video conferencing and one in person). Interviews at work locations promoted a sense of ease by being in a familiar setting and helped participants better recall their flow experiences where they occurred.

### 2.3. Procedures

Author SRC conducted each of the interviews. At the time of the study, he was a doctoral candidate in industrial engineering, with a concentration in human factors psychology (he has since earned his doctorate). In addition, SRC had been a process design management consultant for over 25 years and has collaborated with various back-office transactional functions to streamline operations. He is well-versed in interviewing techniques aimed at establishing rapport, eliciting open dialogue, and probing for relevant information. Participants in the current study were either well known to SRC – having worked together on one or more process design projects – or were well known to individuals who referred the participants to SRC. The interviewer made the participants aware of the purpose of the research and of his academic and professional background. None of the participants had at the time of the study any type of professional relationship with SRC. Informed consent was obtained from each participant prior to the beginning of the interview. After some demographic information was collected, the interviewer read the above paragraph pertaining to typical feelings of flow out loud once again to the participant to focus the discussion on the phenomenon of flow. Then, the following questions guided the discussion, employing the phenomenological interview practices recommended by Lester (1999) and Smith and Osborn (2004):

Describe a work situation in which you experienced a feeling similar to the one just described.
What did that experience feel like?
What triggered that feeling?
To what extent did the challenge of the task influence how you felt?
To what extent did the technology you used influence how you felt?
To what extent did the people you worked with or who were near you influence how you felt?
To what extent did the communication about the task influence how you felt?
To what extent did the physical environment in which you work influence how you felt?
What caused the feeling to end?
For how long do you think the experience lasted?

The participants offered details about each of the topics. The interviewer minimized intruding on the participants’ descriptions of their experiences so as to not interrupt their recollections, nor to introduce interviewer bias into the respondents’ stories. If some aspect of the discussion appeared novel or needed more details to be fully documentable, the interviewer prompted the participants with questions such as, “Can you describe [insert topic] more fully?” and “What else do you think about [insert topic]?” Each interview lasted approximately 45 min.

Interviews were digitally recorded, with permission given by the participants prior to beginning the interviews. Recording allowed the interviewer to be more fully present for the discussion and able to better sense when to move on to another topic or to delve deeper into the current one; and it facilitated an accurate capturing, word for word, of the participants’ experiences for later analysis. The recording device was an Olympus WS-853 Digital Voice Recorder (Olympus Corporation of the Americas., 2021) chosen for its unobtrusive size and sharp recording quality. Once all the interviews were completed, the researcher transmitted the recorded files to a professional transcription company. This company employed human transcriptionists fluent in the language in which the interviews took place (all were in American English). Neither follow-up interviews nor participant reviews of their transcripts were employed in this study.

### 2.4. Data analysis methods

Interview transcripts were analyzed using the methods recommended by Lester (1999), Smith and Osborn (2004), and Leedy and Ormrod (2013). For each question asked of the participants, common themes were identified. Identification of themes was performed by having one of this paper’s authors perform the following steps, as recommended by Belotto (2018). To aid in the search for common themes among the almost 13 h of transcripts, all the transcribed interview files were combined into one master text file (first removing the paragraph beginning with “The task is so demanding and rich in its complexity and pull” since this lengthy text was read by the interviewer at the beginning of each interview and, therefore, should not be included in the transcript text to be analyzed). One of this study’s authors then read through the entire response set. Common descriptions were grouped into themes through a recursive method suggested by Saldaña (2009), pp. 16–19: from the initial transcriptions, themes of flow contributors were identified; as subsequent transcriptions were read, these themes were modified and additional themes were added to more thoroughly and deeply capture the thoughts and feelings being communicated. All transcribed interviews were next re-read to ensure pertinent themes were not missed and those that had been captured were fully documented with all applicable participants’ data.

Although this study is qualitative in nature and no statistically valid sample sizes were used nor were generalized results deduced, the authors decided to adopt a guideline for when to create a particular theme from participant feedback. The heuristic used to affinitize responses into common themes was that three or more participants had to comment similarly on a topic before a theme could be created. The authors believe this requirement lends strength to the method used to generate themes.

The author who performed the initial coding of themes (SRC) then had the other two authors review this categorization to comment on the extent to which the participants’ responses “fit” into the themes suggested by the first author. Changes to themes were agreed upon by all three authors. This method of ensuring reliability is similar to that documented by Belotto (2018).

The investigators were careful not to interpret individuals’ responses during the interview sessions, which may have led to inadvertent insertion of the interviewer’s own beliefs. Rather, the researchers grouped common experiences into themes that captured overall essences of the topics being discussed. Finally, thematic interpretation took place after all interviews were completed in order to match – to the extent possible – these grouped observances with the nine classic dimensions of flow.

**FIGURE 1:**
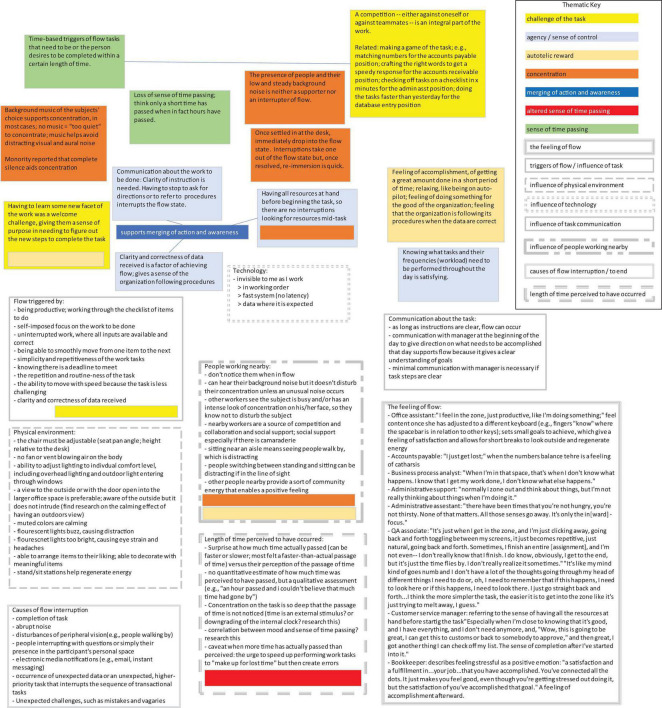
A portion of the thematic map of interview responses categorized into flow dimensions (color-coded boxes) and/or interview themes (gray outline boxes).

Microsoft Excel (Microsoft Corporation, 2018) was used to give structure to participant responses. This application was chosen for its ease of data entry and its use of free-form text boxes in which to enter response data. Responses were grouped into color-coded text boxes according to their common themes. The research questions and dimensions of flow were the themes; there was no exploratory search for themes needed. Therefore, coding systems such as concept maps and coding trees were not used to further identify and arrange the themes, as the researchers determined that the data captured for each theme were self-contained and not hierarchical in nature. The thematic map developed from analyzing the responses is shown in Figure 1.

## 3. Results

The interviewee responses documented in this section correspond to the items presented in the interview guide. Prompts about the flow experience and triggers of flow are described in the sections “3.1. The flow experience described” and “3.2. Triggers of flow.” Since the literature describes the flow dimension of challenge/skill balance as the most critical antecedent for creating flow-inducing conditions, section “3.3. The challenge of the task” is devoted to better understanding the type of challenge presented to the individuals. We especially felt understanding the job challenge was important given the paradigm we were operating under of relatively straightforward work that was being performed. Sections “3.4. Technology’s influence on the achievement of flow” through “3.7. Physical environment’s influence on flow” discuss the participants’ flow experience under the framework of the four types of non-task work system factors under consideration for the present study. We were interested in documenting the conditions under which the “spell” of flow could be broken; participants’ feedback in this area is documented in the section “3.8. Interruptions to the flow experience.” One of the present researchers (PAH) has studied perceptions of elapsed time while in flow (Hancock et al., 2019) and so we were particularly keen on understanding the present set of individuals’ descriptions of the passing of time while in flow; this matter is covered in the section “3.9. Perceptions on the length of time of the flow experience.” A thematic map, a portion of which is shown in Figure 1, was used to document and categorize the findings of this section.

### 3.1. The flow experience described

In discussing the prompts “Describe a work situation in which you experienced a feeling similar to the one just described” and “What did that experience feel like?,” one common theme was that once the subjects sat at their workstations and began their transactional activities, nearly all of them quickly sank into the immersive state they describe as “flow.” That is, once they turned on their desktop computers, arranged their workspaces (to be discussed in a later section), identified the work tasks to be accomplished, and began said work tasks, they easily became deeply focused on their transactions, with particular emphasis on completing them all before their work shifts ended. As a database entry clerk noted when asked to estimate the length of time she needed to achieve a feeling of flow similar to the passage read to her at the beginning of the interview, she responded “Oh, that’s pretty immediate getting into it. It’s pretty immediate.”

Work tasks were transactional in nature: entering data, updating document content, and balancing accounting entries are some examples of the activities performed. According to the participants, this type of work typically requires the quick succession of completing the following sub-tasks:

Identifying all transactions to be completed typically occurs at the beginning of the work shift. This queue of transactions may consist of a set of awaiting emails, a list of items in a database to be processed, a stack of paper documents, or some similar mass of work to be addressed. The office manager, customer service manager, and administrative assistant created paper-based lists of their work-to-be-done; they would pencil through or draw check marks next to their complete items during the day.

The subjects described identifying and gathering resources needed to perform the task as, for instance, gathering all the necessary instructions and operating procedures; accessing the proper computer applications; and gathering test equipment. These resources need to be at hand and functioning properly before performing the steps needed to complete the work task can begin. For example, in the case of instructions and operating procedures, participants indicated these tools must be available, current, and importantly, so familiar that they do not need to be referenced. Likewise, computer applications must work smoothly (including no operating system or network glitches) and intuitively. Test equipment must be fully functional and calibrated to produce accurate results.

Acquiring the next transaction consists of identifying the next task on which to devote time and effort to resolve. Identification occurs through such vehicles as lists of transactions (accounts payable clerk; bookkeeper), an author’s edits on a document (office assistant), and queues of bills of lading (customer service manager). One can think of these items as work orders arranged in a first-in, first-out basis.

Only after identifying the transaction to be completed and ensuring all resources needed to complete that transaction were available and functional were the participants ready to execute the steps to complete said transaction. Once they completed the current transaction, the individuals checked off the task – either mentally noting completion or physically notating the list of transactions – and began the work activity for the next transaction. An office manager (OM), responsible for updating customer payments received by her organization into the general ledger system, described the steps of identifying the transactions to be completed and of notating the completion of those transactions:

*OM:*…*I can print out that whole set [of payments received for] that week.*

*Interviewer: To what extent does the [printed list of payments] help you get into flow or not?*

*OM: The paper does*…*because I can actually mark on the paper, “Okay, so this payment’s been completed,” mark. “This one’s been completed,” mark.*

**FIGURE 2:**
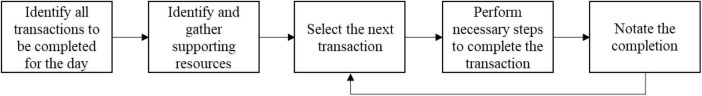
Decomposition of a set of transactional work items into sub-tasks.

According to the subjects interviewed, steps one and two in Figure 2 had to occur for each transaction before those individuals could begin to become immersed in their work activities.

One of the subjects, a customer service manager (CSM) who is responsible for shipping products to overseas customers, described the above set of steps in terms of a sense of completeness when all necessary resources are available for her to execute the shipping order:

*CSM: After I put everything all together is when I have everything in front of me that I need. If I have it, I looked at it, and I’m like, “Okay, what do I need for this?” I might have to walk around and get different things or talk to someone about it or make a phone call. It would be after I started working on it, and then I know [I can begin smoothly and without delay]. Especially when I’m close to knowing that it’s good, and I have everything, and I don’t need anymore, and, “Wow, this is going to be great, I can get this to customs or back to somebody to approve,” and then great, I got another thing I can check off my list. The sense of completion after I’ve started into it.*

*Interviewer: Tell me if I’m hearing this right. It sounds like when you have all your resources together at hand, that’s when you get into that deep immersion state?*

*CSM: Exactly.*

And then, as the CSM completes each shipping order, she stated “In my head, I’m clicking off “Done. Done. Done.” It’s my drive for a sense of accomplishment. It makes me feel really accomplished and valuable.”

Others similarly described flow in terms of a sense of accomplishment. A database entry clerk, responsible for documenting donors’ monetary gifts to a non-profit organization, commented, “It feels fulfilling, satisfying because I’m getting it done. I’m helping us work toward a goal and it’s a good pace.” An office assistant noted, “I feel in the zone, just productive, like I’m doing something.” A bookkeeper related she feels stress while doing her job, but she couched the sentiment in positive terms: “A satisfaction and a fulfillment in your job that you have accomplished. You’ve connected all the dots. It just makes you feel good, even though you’re getting stressed out doing it, but the satisfaction of you’ve accomplished that goal.” A production support specialist, responsible for assigning and tracking the progress of incoming insurance claims, noted, “It makes you feel accomplished because you’re like, ‘Oh sweet. My numbers are really high. I’m really not in doubt. I’m making a difference today.”’ An accounts payable clerk described the phenomenon as “I just get lost” in comparing lists of numbers; and when the numbers balance there is a feeling of “catharsis.”

The sensation of feeling “lost” was repeated by others, in terms of becoming unaware of time passing or forgetting the self. A business process analyst, whose responsibility is to manage employee access to her organization’s systems, related her description of being in flow as, “When I’m in that space (being in flow), that’s when I don’t know what happens. I know that I get my work done, I don’t know what else happens.” An administrative assistant described her flow experiences as, “There have been times that you’re not hungry, you’re not thirsty. None of that matters. All those senses go away. It’s only the in[ward]-focus.” A quality assurance associate, who is responsible for checking that customer service representatives in her firm are interacting with clients professionally and within policy, also noted a loss of self-consciousness as well as performing without consciously thinking of which actions to execute:

*It’s just when I get in the zone, and I’m just clicking away, going back and forth toggling between my screens, it just becomes repetitive, just natural, going back and forth. Sometimes, I finish an entire [assignment], and I’m not even– I don’t really know that I finish. I do know, obviously, I get to the end, but it’s just the time flies by. I don’t really realize it sometimes.” “It’s like my mind kind of goes numb and I don’t have a lot of the thoughts going through my head of different things I need to do or, oh, I need to remember that if this happens, I need to look here or if this happens, I need to look there. I just go straight back and forth. I think the more simpler the task, the easier it is to get into the zone like it’s just trying to melt away, I guess.*

### 3.2. Triggers of flow

When asked what factors helped immerse themselves into the work, most of the participants described their sense of productivity with a linked desire for completing the work as the trigger. For example, one of the office assistant’s (OA) responsibilities was to update journal articles with the author’s edits. She related that setting and accomplishing short-term goals gave her the deep satisfaction of a rewarding experience:

*Interviewer: What do you think triggers [flow]?*

*OA:*…*the fact that I’m getting something done, that I’m being productive, it’s not for nothing. I have some type of goals set and it’s to finish this page. Since I’m working toward that, I don’t want to get distracted by anything.*

*Interviewer: Interesting. It sounds like you set short term goals, finish a paragraph, finish a page.*

*OA: I do. Yes, I have to.*

*Interviewer: Then once you achieve the short-term goal, do you set another short-term goal?*

*OA: Yes, I do.*

*Interviewer: Talk about that.*

*OA: Typically, I look at a task, so he’ll have a lot of pages, maybe 16 pages of revisions. I don’t know how I managed to do it in a short period of time, but I do. I look at the pages and I get– It looks daunting. It’s like, “Oh my gosh, I don’t want to do all of this.” Then I think about it in increments, and with the way I work is in breaks, I have to take breaks. I can push myself but it’s not as– I don’t know. I feel like I need smaller goals just to make it more pleasant, the experience more pleasant, so I’ll take a couple of pages.*

An administrative assistant offered this take on production as her flow trigger: “Scratching off [completed items on a checklist]. I had written things on a piece of paper. As I did five at a time, just the feeling of accomplishment, of being able to cross off a name, because I did that one, and I did that one, and I did that one, and I did that one.” A customer service manager commented, “In my head, I’m clicking off “done, done, done.” It’s my drive for a sense of accomplishment. It makes me feel really accomplished and valuable.” The database entry clerk noted, “A trigger that gets me, I think it’s just my own personal work ethic of got to get the job done and get it done right and get it done fast and don’t want anybody complaining.” And an office manager, responsible for paying the company’s bills, contributed to the theme of productivity and completion: “I think it’s just going from one thing to the next because it’s not just one little section of it. There is probably, say, 50 payments that I’m having to take care of, and it’s just from one to the next, to the next, to the next. I think that’s how I can lose myself in it just because you just finished one, you go on to the next…”

The space utilization coordinator, responsible for assigning physical classroom space to university course sessions, commented on his triggers of productivity/accomplishment as well as having a quiet workspace in which to concentrate:

*Once I have [the course offerings and instructor names], it’s almost as though that’s a peaceful thing for me. Because as soon as I have the list, it’s a sense of calm because then I know, this is what I’m doing, this is what I have to accomplish. The data is right here. All I have to do is get the data from point A to point B. You’re just going through a smooth flow and you just enter it. As long as there’s no interruptions, no one coming into your zone and interrupting that flow, I’ve been known to go for hours at that point.*

Others also commented that when they are able to concentrate on the work to be done, they experience flow. The accounts payable clerk said, “Okay, at twelve o’clock I’ve got this meeting, so it’s a little bit hard to get in that zone, but it’s like if I know [that the work is] what I’m concentrating on, I’ll jump right in and just sit there.”

### 3.3. The challenge of the task

When asked to discuss the challenge of their work as a proponent of flow, many participants commented on the relative ease of executing their tasks. The lab analyst, whose role was to analyze chemical components of a consumer product line, noted, “I like the pace, so I didn’t want a big job.” The administrative support clerk, responsible for a number of tasks, responded to this prompt, “For this (timesheet entry task), because I already know, and it was going to be pretty easy, and I know who to email, that there’s no stress with it, so it’s kind of, not necessarily relief, but kind of like a break almost because I need to do this (task) every … other week, but that’s time where I know it’s going to be fine really.”

Also, for many of the participants – and related to the theme of execution ease – was the theme of each task item being similar to the previous one, resulting in a lower level of task challenge. The office manager responsible for fulfilling customers’ shirt orders, said, “It’s pretty automated. I get into a routine, I put the stuff in the bag, and then I weigh it and I write it down and then I go onto the next one. There’s like a process.” The business process analyst echoed the theme of sameness regarding her work on updating system access permissions for hundreds of employees:

…*even though there are a lot of steps to it, it’s not necessarily challenging*… *(each work item’s steps are) one, two, three. There is no deviation. There are steps that have to happen in order. I made it my job to learn all of these different steps in order so that way I can focus. It’s not challenging. In the very beginning, it was challenging but I got it to where I know everything that I have to do and I think that made it easier for me to get into flow state because now, it’s kind of autonomous. It’s automatic in exactly what I need to do. It’s very easy for me to complete it.*

The space utilization coordinator echoed the theme of repetitiveness:

…*if it’s a new task that I have not performed, I do not have my general modus operandi. If I don’t have that all set up in my head, it’s more stressful, so therefore I don’t feel as though I relax and get into that flow as quickly. The first time that I’m doing something, or if it’s a new job function, I would not say that I get into a flow. Once this is something that I’ve done before, I have seen the result of it, and that the result was correct, because you have to actually be able to see that what you did the first time was a correct action or result, and that means you can keep repeating that same work and come to the same conclusion. As long as I am not doing something that’s new, I can get in the flow, but if it’s something new, that I find that stressful and I don’t get into the flow as quickly, until I repeat that, say once, twice, three times until I’m comfortable.*

A quality assurance associate, who has the responsibility to check customer service representatives’ recorded calls for compliance with company, state, and federal regulations, responded to the question of task challenge as a combination of ease and repetition:

*I feel like maybe it’s the simplicity of [the work] and the repetitiveness of it because it’s just very simple repetitive tasks. I just go to one screen, get the information I need, see if they did it (completely complied with regulations), and then I go to my database, write a document if they passed or failed, and then hit complete and I do it all over again. I just go back and forth*…*I feel like if it’s simple, I don’t really have to think about much. It’s like my mind kind of goes numb and I don’t have a lot of the thoughts going through my head of different things I need to do or, oh, I need to remember that if this happens, I need to look here or if this happens, I need to look there. I just go straight back and forth. Did it happen or did it not? I think the more simpler the task, the easier it is to get into the zone like it’s just trying to melt away*… *If it’s complex, I have to use my brain. [laughs] If I do that, then I’m triggering memories and conversations*…*thinking about the emails that I’ve read to make sure that I’m doing what I’m supposed to do. Like those what-if scenarios because our tests are like that or they’re more complex. If they did this, but it’s wrong, but it may not be wrong if this applies or this applies or this applies. Trying to think back to conversations I had and if it’s applicable in emails or communications that might have come up.*

A second quality assurance associate, working in the same organization and with the same responsibilities as the first associate above, commented similarly: “…because if you’re flowing along, and you’re doing your daily routine, and you’re not necessarily having to get out of the box, so to speak, and you could lose track of time there, too, because things are flowing, you’re trying to (meet your productivity goals), and you’re going along.”

Another theme described by the workers was in creating goal-oriented challenges for themselves. For example, the administrative assistant said.

*I’m not allowed to be here after five, and I find myself looking and it’s 4:49, and I’m like, “How much can I get done in 11 min?” And you challenge yourself, but you need to be careful and mindful, so you’re not creating errors, because then, of course, that’s time taken away that you need to fix. But I challenge myself. I look at the time*…*or set an alarm, or something, or if someone says, “Goodbye.” I know it’s that time of the day (to leave). So, I guess I like to challenge myself in a timeframe. “How much can I get done? Can I get to this part of the (task) list?”*

The database entry clerk also described a time-based goal-oriented trial: “I challenge myself. I know how fast I may have done the same task the day before, I try to do it even faster. It’s like I play games with myself and I change it up for myself. It’s a competitive thing against myself.” The business process analyst stated that when she enters lists of newly hired employees’ system information into a database, she challenges herself to perform the data entry as fast as possible:

…*if I get*…*a list of say 30 employees that I’ll have to hire on as a new hire. I’ll have to log into a system and I know how many clicks, how many steps it takes to get into that system. I know exactly where on the computer screen that my mouse will need to hover over, so I know exactly what to do to (complete) that whole process. If I can onboard 30 people in the most amount of time, and so what I like to do is make it a game. I’ll try to get very precise each time that I click on somebody because it’s the same repetitive motion over and over again 30 times until I get through all of each person one. I’ll have to do all of these different steps to get to person two, so I’ll try to make it a game to see how quickly I can get through that. Just going back and clicking and just kind of losing that sense. Getting through as many of them to make sure that I don’t miss anybody and that all 30 people had everything that they need by X amount of time.*

A similar sentiment was offered by the space utilization coordinator:

*In terms of if you can challenge yourself mentally*…*you can say, “I can complete this by X time of day,” or, “I think I can do this in 3 days. You know, last time I did this list, I did this in 3 days, maybe I can do it in two.” Sometimes I set up little goals for myself mentally. I don’t always hit them, but it’s just a point of having them or giving yourself a goal to strive for, so basically a temporary little goal you set up within your mind, so that instead of, “Well, I’ve got 140 different entries to make today.” It’s more of an aspect of, “How quickly can I get those done?” It becomes (an) efficiency sort of equation in my mind, versus, “Here we go, it’s a drudgery,” kind of a thing. It’s a mental mindset.*

While the above challenges were inward-focused, other study participants created outward-focused challenges. These challenges took the form of competitions against other teammates. For example, the production support specialist strove “have the higher numbers (completed work items)” than her co-workers, and where almost everyone would “stay 2 or 3 min longer just to get the higher number.” The lab analyst stated that her site was continually challenging itself against another site to complete more chemical assays: “…we’re doing more (work assignments than) even done in (the other work location).…we need to be better, yes, it was a competition with (the other work location).”

Many of the interviewees listened to music while working. Although most of these individuals used music as a means of tuning out distracting office sounds to support their ability to concentrate on their work tasks, two engaged their musical preferences in a novel, self-imposed, and inward-directed challenge to perform their task steps in time to the musical beat. As the accounts payable clerk noted.

“…*I make it into a game. I try and make it fun as much as I can*…*with the music and the cadence*…*It was like The Cars could be on or some other group could be on, Lynyrd Skynyrd or ZZ Top and I’ll sit there with the music and the beat will be one, two, three, four or something. I’ll be like, five, six, seven. Two, three, four, and I’ll just go to the beat and just see if I can do little tempos and beats to make (balancing the accounting numbers) a little bit more fun.”*

The business process analyst likewise recounted, “(I) put my music in and get into a rhythm and get all of my tasks done.”

### 3.4. Technology’s influence on the achievement of flow

Non-task variables, or work system factors, have been hypothesized to combine with the challenge of the task and other task-centric flow antecedents to create the conditions for an optimal experience, as described earlier as the work system theory of flow (Clapp et al., 2018). Sections “3.4. Technology’s influence on the achievement of flow” through “3.7. Physical environment’s influence on flow” document the influence of the present study’s selected non-task work system factors on participants’ ability to achieve flow.

The common technology theme from the interviewees was that the set of work tools had to become invisible while using them: the tools were expected to work well each time they were accessed so that no thought was expended on wondering if those tools were performing as needed. For example, one of the office assistants interviewed discussed her desire to use a comfortable keyboard:

*If I have a keyboard that’s more comfortable to use, it makes it easier on my hands so that I can spend more time focusing on the work, on the words. I’ll use my dad’s computer to do some other work, but his keyword is very old, and you have to press the buttons really hard, and so you’re focusing more on, “Did I type that correctly?” versus when you’re typing*…*you’re not really looking at the keyboard (but you’re) looking at the paper, because you can feel, “Okay, I know I’m typing this correctly.” It’s less broken up. The process is less interrupted by, “Did it [the key press] really go all the way in for the letter to show up on the screen?”*

The business process analyst described a task that requires copying data from spreadsheets into a computer system. Various personnel in her organization email her these spreadsheets. If one of the spreadsheet’s columns of data are not in the expected order of occurrence – which should match the order of copying and pasting – she must first rearrange the columns to comply with the correct sequence. Only then she can perform her work task with rhythmic ease:

*Sometimes, whenever a spreadsheet comes over and it’s not exactly in the order that I need it to be, I’ll be able to quickly recognize what order it needs to be in. Shift some stuff around, a couple of things around, and then I can toggle back and forth, copy-paste all of the information. It’s a lot of copy-paste from the keyboard. It’s a lot of the keyboard shortcuts that I know*…. *I’ll take the information from the Excel spreadsheets, toggle to the other system, pasting information where I need to. I know exactly how many times that I need to hit the tab key to [arrive at the fields where I paste the data].*

The space utilization coordinator, whose responsibility is to assign university classes to physical and virtual classrooms, commented on how slow-moving technology prevented him from achieving a data entry rhythm and precluded him from experiencing flow:

*The system pauses for a second. If it takes longer than*… *3 or 4 s, then my routine is messed up, because then I hit the tab key because then the next thing I’m doing is I’m entering the start time, and then I’m entering the end time. I normally have a flow. You get a flow going. I know that this takes about this amount of time. My muscles remember and they say, “Okay, we’re going to hit that tab key at this point in time, you’re going to then go to your number keys, you’re going to type in your time. You’re going to hit the tab key again, you’re going to enter your end time, and you’re going to tab out. Then you’re going to move down to instructor, you’re going to click.” You have your muscle routine there*…. *If you then have your software, where it decides that’s going to be slow, or the servers are down, or what have you, then it takes five, seven, God forbid, it used to take sometimes up to a minute, just to load what you did. You can’t proceed until you enter one thing, it does this little swirl and then it says basically done, and then you can move on, because if you enter other stuff, it just wipes out (the previously entered data) when it updates.*

### 3.5. Co-workers’ (background noise) influence on the achievement of flow

According to a few of the subjects interviewed, the nearby presence of co-workers can support the achievement of flow under certain circumstances. The low background noise generated by an office full of people helped the present study’s subjects concentrate on their own tasks; the bookkeeper noted that because she raised five children with their attendant noise, a silent office “…[is] too quiet…and I can’t sync as well. The background noise is helpful.” Another condition where co-workers’ presence aids in the acquisition of flow is when those working nearby share a sense of camaraderie with each other. Such amity helped create a sense of ease, belonging, and community energy and community productivity according to many of those interviewed, and the relaxed atmosphere supported their ability to concentrate on work tasks.

Nearby co-workers can hinder the occurrence or continuation of flow if they create unexpected clatter, noticeable above the low thrum of background noise. Loud laughter or the scraping of a chair can contribute to interrupting concentration, which the interviewees described as contributing to an interruption in their immersion. Being in the line of sight of others moving about also interrupts concentration. The quality assurance associate noted that when she was located in a cubicle adjoining an aisle, noticing those who walked past her interrupted her focus on the work and prevented her from experiencing flow. However, the playing of music in headphones described in the section “3.3. The challenge of the task” mitigated to some extent those interviewees who used this device from losing concentration.

### 3.6. Task communication and its influence on flow

With an average in-role tenure of over 7 years, supervisor instructions on what, when, and how to perform the work was not required from the study’s subjects. Referring to standard operating procedures was also generally not needed. However, when some aspect of their work changed – say, a technology upgrade that modified the workflow or a newer set of customer requirements was introduced – then this study’s individuals commented that they needed to consult with their resources for a period of time to ensure understanding of the modified work. As the quality assurance associate remarked, “I would have a lot of stopping points to go and ask questions like, ‘[Am I] I really looking at this right or how do I do this, where do I go to find this?”’ Until they could once again become comfortable with the new steps and any work system changes, they found experiencing flow difficult.

Thorough and sensible training and operating procedures were remarked upon as critical for one to become comfortable with work tasks, which then supported flow. As an administrative assistant commented upon learning she had not been fully trained on all steps needed to compete a particular repetitive work assignment, “Being taught something and not 100% knowledgeable on what you’re doing isn’t a good feeling.” Thinking she had successfully completed this work assignment, only to be informed that she had left out a crucial step, “brought my happy high down.” The customer service manager commented how a lack of communication proffered when procedures change results in anxiety, which prevents flow from occurring: “I can sometimes get frustrated with the lack of communication because the sales department isn’t talking with the operations department. Then I’ve already done something, and because they’ve made some changes that I wasn’t aware of because they didn’t inform me, I either have to redo something, or it affects what I’ve already done. That can be frustrating for me.” The database entry clerk (DEC) offered nearly the same feedback on the question of the influence of communication on achieving flow:

*DEC: I think sometimes part of the problem for me with the lack of communication, that does occur a lot, is that sometimes I don’t know if I’m doing it right and I don’t like doing something and then having to go back and change it on a regular basis because I would rather be told properly the first time or communicated with, which that doesn’t always happen so then I get very frustrated if I have to go back and redo things.*

*Interviewer: If communication is not forthcoming, does that take you out of that flow feeling?*

*DEC: Yes, because then I got to stop and go back and redo.*

### 3.7. Physical environment’s influence on flow

This study’s participants commented that a number of physical environment factors did deter from or contribute to their achievement of flow while working. For example, the office assistant, whose responsibilities include incorporating edits into her supervisor’s journal article drafts, related how sitting in her supervisor’s office chair while working on his document is not comfortable. She will swap his seat for hers (they share a physical office space) so she does not have to spend time making seating adjustments. She also shuts off a portable fan on her supervisor’s desk, which makes her cold. Then, “the physical side of things doesn’t get in the way because…I’m not cold, I’m not scooting in. I’m really just focusing on typing.”

Seating discomfort was mentioned by a number of participants. The customer service manager and the database entry clerk utilized a sit-stand workstation, where the desk could be raised to a height comfortable for use while standing, to alleviate back pain and support concentration on the work tasks. The database entry clerk also noted that switching from a seated to a standing position “wakes me right up to be able to stand up. I start getting my energy back when I can stand.” The administrative assistant echoed this sentiment.

Many of the participants noted how a view of the outside and its attendant natural light provided a calming effect. As the lab analyst described the feeling:

*When you have access to see outside, you don’t feel like you are like in four walls*…*. I was seeing*… *the trees, the garden, sometimes some birds in the lake. I don’t know why, but I [felt] less stress. Just seeing how sometimes the [scenery] moves. It was like I [felt] relaxed. Everything was so white, so bright. I [felt] like I [saw] the sun. [Another time, it] was raining, I saw the rain. I think that makes me feel like you’re working, but you have a view.*

The quality assurance associate also commented on how being able to see outside supported a feeling of well-being: “I love the view because it makes me feel relaxed and comfortable. It’s like if I could see like pretty blue skies or a storm going by or whatever, I don’t feel like I’m trapped in a box. The view’s a huge thing for me to feel comfortable.”

Two cautionary characteristics of an outside view noted by a few of the interviewees were that at certain times of the day, sunlight streaming through windows caused glare on computer screens and raised ambient temperatures to uncomfortable levels. Such phenomena contributed to eyestrain and drowsiness, respectively, both of which hindered concentration. The presence of blinds allowed these individuals to control such conditions and create a more comfortable physical environment.

Also supporting a feeling of relaxation and comfort was being able to decorate and customize one’s work space. Such freedom to personalize one’s workspace and provided a sense of the familiar. The accounts receivable manager hung family pictures and awards on his wall to personalize his space. He found being able to see such meaningful items while working gave him comfort. The customer service manager had a number of green plants around her office. She stated the flora served to bring the outside to the inside and provided a relaxing view.

Fluorescent lighting was a topic addressed by a number of participants. For some, these light fixtures produced a brightness – in the words of the production support specialist, “the light of a thousand suns” – that caused eye strain which led to fatigue. The accounts payable clerk also was susceptible to the buzzing sound her fluorescent light fixtures made; she found the noise extremely distracting and used music played through headphones to overcome this disrupting phenomenon.

### 3.8. Interruptions to the flow experience

Factors that caused participants in the present study to exit flow were almost exclusively centered in three areas. Two of those factors were considered undesirable: distractions and unexpected changes in work tasks. The natural conclusion to the queue of work tasks was considered a desirable conclusion of the flow experience. Distractions included co-workers entering one’s workspace to chat or ask a question, email notifications, and loud noises either inside or outside the office. Unexpected changes in work tasks included the appearance of a more difficult item in the work queue, requiring the individual to stop executing and perform some type of research on how to proceed; and the reprioritization of work tasks, usually by one’s supervisor.

However, once those interruptions were resolved, most participants reported being able to re-enter their flow states almost immediately as they resumed their work tasks. The restarting of their personal work cadences helped them regain the feeling of immersion into their work.

### 3.9. Perceptions on the length of time of the flow experience

Most of the present study’s participants did recall their perceptions of time passing were altered. They thought less time had expired than had actually occurred, and expressed surprise when they made themselves aware of the actual passage of time (“I lost track of time” was a commonly repeated experience). For example, the accounts payable clerk remarked, “Sometimes I think it’s only maybe, 5 or 10 min, maybe 20 at the most. There have been a couple times where I’ll think, ‘I’ve done three pages of checking [numbers in a ledger]. It’s probably only been 30 min or something.’ The next thing I know, it’s been almost an hour.”

The administrative support clerk said of this topic while describing a proofreading task, “…I’m just focused on the next sentence, the next sentence I’m reading. I lose track of time a lot. …you get lost in what you’re reading…”

## 4. Discussion

The preceding section documented the participants’ lived experience of flow. Now we interpret these findings according to the classic nine dimensions of flow. The thematic map shown in Figure 1 was employed to translate the themes identified in section “3. Results” into the flow antecedents, characteristics, and consequences.

### 4.1. Low-challenge simplicity and predictability lead to flow

We can compare the transactional worker participants’ descriptions of their flow experiences to descriptions similar to others engaged in more creative endeavors cited in previous research (Csikszentmihalyi, 1988, 1990, 2003; Csikszentmihalyi and LeFevre, 1989).

The extant research on flow deems the challenge/skill balance – that is, the demands of the task should just exceed the knowledge and abilities of the task performer – as the primary driver in creating flow in individuals (LeFevre, 1988; Csikszentmihalyi, 1990; Bakker, 2008). In the present study, where the rote and repetitive nature of the work being performed seemed to constitute low-challenge activities, the researchers were interested in understanding the role of the tasks’ demands in fomenting flow.

Most of the interviewees commented that the repetitiveness and routineness of the work are their flow triggers. The fact that their work tasks are less challenging, which permits a relatively quick pace, is what facilitates the immersive feeling in these individuals. This expectation of task sameness appears to be in contrast to descriptions of flow of those in more creative exercises (e.g., rock climbers and solo trans-ocean sailors; Csikszentmihalyi, 1988, 1990). In these descriptions of flow experiences, the challenge of reading a never-before-climbed rock face to find the right fissure onto which to insert fingertips and of constantly interpreting changing weather conditions to determine how to correctly set the sails were what triggered flow in these individuals.

One possible explanation for how flow can be experienced with low-challenge and continually repeated tasks may be found in the research on the differences between so-called “exciting flow” and “relaxing flow” (Apter, 1992; Chang et al., 2020). These researchers propose that behavior-oriented individuals prefer excitement of and the journey through the task, rather than the predictability and results orientation of goal-oriented individuals. Behavior-oriented individuals tend to enjoy exciting flow, while goal-oriented individuals gravitate toward relaxing flow. As the participants in this present study repeatedly spoke of their desire for similar, repeatable work tasks and their enjoyment in the checking off their completed work, the theory of relaxing flow in transactional workers may be supported.

Ceja and Navarro (2017) describe a smooth pathway to achieving flow as part of their cusp-catastrophe model. Here, low-challenge tasks requiring low skills to competently execute them are the requirements for attaining a flow state. The present study’s transactional workers consistently described their desire for familiar work items in which they knew from much experience how to complete, supporting this theory of flow achievement. We contend that this smooth entry into flow results in the relaxing flow previously described.

### 4.2. Fun constructs support goal-setting and performance feedback

Introducing a dimension of fun into their work appeared to aid at least some of the present study’s individuals in experiencing flow. Self-created and -directed games noted by the participants included challenging themselves to produce a higher quantity of work outputs than other teammates, or to improve on their own productivity from the day before, or to complete each work task faster than the previous one(s), or to execute keystrokes in time to the rhythm of a song that happened to be playing at the time, or to even employing checklists from which they could cross off completed work (this last mechanism provided a means to “keep score”). In addition to creating fun at work, these game-like mechanisms also served to create goals to meet and to provide immediate performance feedback to the workers (e.g., “How many keystrokes can I enter in a row in time to this song playing in my earbuds?” and “Does the rhythm of my pressing of the keys match the beat of the song?”). Unknowingly, these workers were creating flow conditions. As Schaffer (2013) noted, “Designing for flow is important for internal business applications, like a system used by bank tellers or people working in a call center. Finding meaningful challenges and getting clear feedback about progress on those challenges is the best way to make even boring or repetitive work more like an enjoyable game.”

### 4.3. Invisible technology supports action/awareness merging

Using job-supporting tools need to be without thought, similar to the flow concept of merging of action and awareness. The present study’s interviewees were more inclined to be able to work without interruption of thought or action if all the technological and non-technological resources were available and fully functional. This condition supported the ability to deeply concentrate on the work. Upgrades to technology hindered achieving flow until the individuals learned the changes well enough that the technology could once again be used without thought.

Thorough and clear training and standard operating procedures obviated the need for constant communication from supervisors and other resources on what and how to perform transactional work. The less that transactional workers needed to stop their work to confer with others on how to execute task steps, the easier they found themselves getting into and maintaining a work rhythm. Gaining full knowledge of task execution requirements supported workers’ ability to work without conscious thought.

### 4.4. An ability to maintain focus

Participants in the present study described themselves as being able to focus exclusively on their work tasks and as accomplishment oriented. Once the participants readied the resources needed to complete their work items and began to execute, achieving flow was nearly immediate. Even when interrupted by co-workers wanting to talk or by unexpected work tasks, re-immersion into a flow state was quick once the interruptions were resolved. This ability to maintain focus over relatively long periods of time are covered in the state-based versus trait-based theories of flow (see Moneta, 2021, for an overview). That the subject individuals consistently reported their ability to concentrate on their tasks at hand rather quickly and to maintain such focus until their tasks were completed, the trait-based explanation of flow seems to be reinforced. Further, the concept of individuals possessing an action-state orientation – an individual’s ability to maintain focus on a task until its completion (see Barthelmäs and Keller, 2021, for a summary) – is amply described in the present study’s interviews and also supports the trait-based bias toward flow.

### 4.5. Agency in a transactional work setting

One might conclude that a minimal “feeling of control over actions and their consequences” (Moore, 2016) exists in workers in a transactional environment, where the work tasks are routine and their execution codified in numbered steps. While individual work items were in fact standardized, the individuals taking part in the present study offered a number of ways in which they could exercise agency at a more macro level. Choice of seating and the ability to sit or stand while working were two examples of ergonomic control the individuals applied. Individuals could also choose to an extent their office décor – photographs and plants, for instance – to suit their tastes. Work breaks, allowing the individuals to recharge physically and mentally, were self-scheduled. The playing of music through headphones was their choice (more on music playing in the section “4.6. Losing oneself in the work”). These personalized choices may help prime these individuals to feel as if they have some measure of control over their work – or at least their non-task work system [as described in Clapp et al. (2018)] – when they arrive at their offices each day, and provide a sense of free will in performing their work roles. Per Moore (2016), both of these variables support the feeling of agency.

### 4.6. Losing oneself in the work

Interviewees in the present study commented how they found themselves working through lunch and biological breaks. Hunger and the need to eliminate were subordinated to the task at hand. Similar behavior has been described by Csikszentmihalyi (1990) of artists completing their works without pausing. Of course, such behavior may be harmful and microbreaks should be encouraged.

These individuals also noted how, while listening to music when working, they would tap their feet or dance, actions they may not perform if they realized at the time their neighboring workmates could see and hear them. The authors surmise that although the simplicity and repetitiveness of the work permits an easy submersion into the task, music may play a role for some in elevating mood (see Wesseldijk et al., 2019, for an overview of the relationship between music and mental health), which may be expressed via rhythm-keeping actions. Since flow has been correlated with positive mood (cf. Fullagar and Kelloway, 2009), music playing as described in the present study may serve to enhance the flow experience.

### 4.7. An altered experience of time

Some of the present study’s participants commented on unknowingly working through lunch or breaks. These workers found this altered perception of time pleasurable as the occurrence of the phenomenon usually resulted in more time passing than expected, which helped them feel as if they accomplished more work in a shorter period of time.

### 4.8. Simplicity and predictability are components of an optimal experience

Participants in the present study translated the quick pace enabled by the simple and predictable tasks into a feeling of being productive, which they indicated was a flow trigger. They offered that they felt a sense of accomplishment as they checked off each work item as complete. The authors offer that the flow-triggering feeling generated by being productive may have been conflated with what has been described in the literature as an autotelic experience by Csikszentmihalyi (1990), especially since productivity is an outcome of the various factors the interviewees noted as necessary for such an experience. For example, the work items must be reliably similar to each other and easy to perform; all supporting resources must be available and functional; and non-work system factors must be at acceptable settings. Relatedly, the autotelic experience is an outcome of the antecedents and characteristics described in the introduction to this present paper.

### 4.9. Comparisons to conventional flow dimensions

This paper has deeply delved into the transactional flow experience as lived by the 17 interviewees. We now want to map the essence of these experiences to the nine conventional dimensions of flow developed through the study of individuals acting in more creative endeavors (Csikszentmihalyi, 1990, 2003; Jackson and Marsh, 1996; Chen et al., 1999; Fullagar et al., 2017; Clapp et al., 2018). See Table 1 for details. We make this comparison to demonstrate that the relaxing flow of transactional workers is similar in construct to the exciting flow of those who can exercise more creative agency in their task execution (Apter, 1992; Chang et al., 2020).

**TABLE 1:** A mapping of interviewees’ described characteristics of their transactional flow experiences to conventional flow dimensions.

Time-ordered position	Flow dimension	Examples of characteristics provided in present study
Antecedent (pre-flow)	Challenge/Skills match	Simple and predictable tasks support the goal-oriented-challenge (that is, “relaxing flow”) needs of transactional workers.
	Clear task goals	Workers establish game-like targets to meet (e.g., creating task lists of work items to be accomplished each day; setting productivity targets, either to improve one’s own productivity from a previous work session or to execute more tasks than another team; attempt to work to the beat of music currently playing on headphones).
	Immediate feedback	Workers physically checked off task list items as they were completed, providing a running source of feedback. Productivity targets were checked against actuality using these checklists.
Characteristic (in flow)	Action/awareness merging	Supporting the ability to execute without conscious thought are clear instructions, complete and correct data, and all tools needed to complete the task available to the individual.
	Intense concentration	Once settled in at their desks with all resources at the ready, the interviewees were able to deeply focus on their work. Passersby and ambient noise did not interrupt their concentration, unless such factors were overly loud. For many of the subjects, background music of their choice supported concentration by masking distracting noise. A trait-based propensity for deep concentration until a task is completed may also support the ability to concentrate for long periods of time.
	Control/sense of agency	Macro-level agency over non-task work system factors, rather than control over how the work is to be performed: individuals elected when to take work and lunch breaks; chose to sit or stand while working; chose the most comfortable seats available in the building for their office; played mood-enhancing music; and had the latitude to decorate their workspaces much to their liking to create a pleasurable surrounding.
Consequence (post-flow)	Loss of self-consciousness/sense of self	Workers forgot to take meal breaks or to take bathroom breaks until they emerged from their flow states. They danced or tapped their feet in time to the music they were playing with seeming disregard for their neighboring workmates watching.
	Altered sense of time passing	Many individuals noted that more time had actually passed during the day than they had sensed. Some worked through their lunch breaks without realizing they had done so.
	Autotelic motivation	Intrinsic motivation included a feeling of self-accomplishment when a great amount of work was completed in a short period of time, and when more work was accomplished than on the previous day or faster than during the previous day; and a feeling of relaxation.

## 5. Limitations and directions for future research

This study does have some limitations. As phenomenological studies are by nature qualitative, the sampling purposive, and the sample size relatively small, no generalizations about flow in transactional workers have been or should be made. However, the present research uncovered some interesting aspects about the transactional worker flow experience revealed in multiple participants’ responses. These factors and feelings should be quantitatively studied through controlled laboratory studies. The objective of such research should be to incorporate non-task work system factors into the overall design of work to encourage the onset and maintenance of flow, given its well-documented beneficial results.

A related limitation of the present phenomenological research is that the interviews were guided based on the research problem the researchers are addressing. The present study attempted to understand what flow feels like for the interviewed transactional workers in the context of the influence of five work system variables thought to be commonly extant in transactional office settings on the occurrence of flow: the challenge of the work tasks; the technology (resources) employed to support the execution of the work; the background noise generated by the people typically co-located with the participants in the office; the level of detail inherent in the communication given about the work tasks; and the physical environment in which the individuals work. Discussion was limited by this scope (and by the time considered reasonable to ask individuals to postpone their work and participate in this study). There certainly can be other factors not introduced in this present research but ultimately important to the launch and sustainment of flow. Future studies may include additional work system factors, with the results broadening the understanding of the transactional work flow experience.

It has been said that interpretive phenomenological analysis (IPA) attempts to deeply understand a particular experience with open-ended questions and little prompting by the interviewer, in order to get at the complete who/what/when/where/why/how of a thing (Smith and Osborn, 2004; Finlay, 2009; Belotto, 2018). An argument may be made that the present study’s interview items that asked participants to comment on the prompts “To what extent did xxx influence how you felt?,” where “xxx” was the challenge of the task or the non-task work factors being researched, may have violated this principle of openness and expansiveness by restricting the conversation to particular topics. Belotto (2018) discovered, however, that the interpretive approach permits the researcher to explore certain specific suspicions and theories that support the overall research question. Smith and Osborn (2004) support this expanded view of IPA: “Do I have a sense of something going on here that maybe the participants themselves are less aware of?” Finlay (2009) expressed support for a number of phenomenological approaches along a continuum between description and interpretation, and that flexibility in how a thing is to be understood is encouraged. We contend that, while we began the interview with open-ended prompts (“Describe a work situation…” and “What did that experience feel like?), we were particularly interested in the impact that the holistic work environment played in creating flow conditions, so we necessarily had to direct the conversation so the interviewee could reflect on and discuss specific non-task work dimensions. However, the dimensions were broad in nature – technology, people, communication, and physical environment – that we feel comfortable in our elicitation approach that we did not limit the interviewees’ ability to talk at length about their flow experience.

The interview responses, once transcribed, were manually reviewed for common themes. As thorough as the researchers believed they were, it is possible that themes were missed. Future researchers may want to analyze the transcripts of the present study and new studies using language processing software. Such tools can quickly extract meaning and identify keywords in context to create a rich dataset.

Once the interviews were transcribed, no further contact with the participants by the researchers were made. Additional experiential information may have been able to be gleaned by having the participants review their transcripts and the authors’ findings, and provide comments, amplification, and correction, as recommended by Tong et al. (2007). Future phenomenological studies should consider using this second-interview approach to potentially gain additional insights into the study topic.

Only those transactional workers who experienced flow were interviewed for the present research. Future studies should attempt to discover the proportion of the transactional worker population who regularly enjoy the flow state, as it is presently unknown to what extent this phenomenon occurs in the subject population. These future studies should also investigate why some transactional workers may not experience flow. This knowledge about flow-ers and non-flow-ers may assist organizations in designing non-task work system factors to support the occurrence of flow in its workforce.

## 6. Summary

This study attempted to document the lived experience of psychological flow in transactional workers through in-depth interviewing of 17 of these individuals and careful analysis of their responses. Although their work tasks are rote and repetitive, the study participants appeared to experience relaxing flow. A possible explanation for their enjoyment of this type of flow (as opposed to exciting flow) may be due to these individuals’ expressed desire for accomplishment (as opposed to the process of the journey). Participants found ways to create their own challenges supplemental to the work itself through devising games for themselves that would help make the work more fun. Check-off lists; competitions against others or against themselves to produce more or to produce faster; and entering data in time to music all supported an enjoyable experience.

Relaxing flow shares the same concepts as traditionally studied exciting flow. Portions of the experiences described in the participant interviews were easily mapped to the classic nine flow dimensions described in the literature. This mapping supports the present study’s contention that flow can be and is experienced by transactional workers. Further research should be aimed at creating working conditions that are conducive to engendering flow in transactional job roles.

## Data availability statement

The raw data supporting the conclusions of this article will be made available by the authors, without undue reservation.

## Ethics statement

The studies involving human participants were reviewed and approved by the University of Central Florida IRB. The participants provided their written informed consent to participate in this study.

## Author contributions

SC, WK, and PH contributed to the conception and design of the study. SC conducted the study and prepared the first draft of the manuscript. All authors contributed to the manuscript revision, read, and approved the submitted version.
